# Impact of COVID-19 on lifestyle and financial behaviour: The implications to research in financial vulnerability

**DOI:** 10.3389/fpsyg.2022.1073017

**Published:** 2022-12-13

**Authors:** Ning Ma, Yam Wing Siu, Tsun Se Cheong, Brian Tung

**Affiliations:** ^1^Hainan College of Economics and Business, Haikou, China; ^2^Department of Economics and Finance, The Hang Seng University of Hong Kong, Hong Kong, Hong Kong SAR, China

**Keywords:** personal finance, digitisation of the economy, COVID-19 pandemic, addiction to digital technology, financial vulnerability

## Abstract

The outbreak of coronavirus pandemic in late 2019 posted unprecedented social-economic challenges and disruptions to societies and individuals. The “new-normal” styles of living and working could intertwined with other determinants complicating the investigation of individual’s financial vulnerability. The purpose of this paper is to conduct literature survey to review and consolidate the recent scattered literatures to identify some possible factors to be considered in the research related to financial vulnerability, including pandemic’s impact of COVID-19 to different aspects of personal finance issues, pandemic-driven digitisation of the economy activities, changes in financial behaviour and addiction to digital technology.

## Introduction

The outbreak of coronavirus pandemic in late 2019 has not only brought about health impacts worldwide. Lockdown and the measures to restrict social and economic activities have adversely affected both the business community as well as the financial conditions of individuals and households. The impacts of the pandemic on personal finances should not simply be attributed to the rising unemployment and declining household income. The COVID-19 pandemic has posted unprecedented social-economic challenges and disruptions to societies and individuals, leading to some other possible determinants of personal financial problem caused by the “new-normal” styles of living and working.

The above-mentioned new challenges and complicated socioeconomic changes in the recent years have made the root causes of financial vulnerability more difficult to analyse because there could be some new determinants of personal financial problem caused by the “new-normal” styles of living and working, such as more rely on digital services for consumption, entertainment and banking. At this moment, research covering those issues were scattered. There are very limited research covering all these issues holistically. It is necessary to spend effort to summarise the possible newer determinants of financial vulnerability of peoples.

The objective of this paper is to look into some relevant but scattered literatures in the area of financial literacy, pyshcology and consumer behaviour to synthesise prior works to identify factors deserved investigation due to the lifestyle and financial behavioural changes brought by the outbreak of COVID-19 pandemic, so as to create value and contribute to body of knowledge of personal finance and financial vulnerability.

## Literature survey

### Financial vulnerability

Financial vulnerability refers to individual’s capacity to cope with adverse financial condition or the perceived feeling of being in a financially unstable situation, representing an early indicator of financial stress (European Credit Research Institute (ECRI) and Personal Finance Research Centre (PFRC), 2008; Klasen and Povel, 2013; de Clercq et al., 2015). Van Aardt et al. (2009) developed a heuristic financial vulnerability model concluding key factors leading to financial vulnerability, such as income fragility, expenditure, saving/income, consumption and debt.

After the model was built, which has categorised factors into base elements, many variables identified during the model development phase were not systematically analysed. More than 10 years later, some of these variables that might be less relevant then are now deserving re-investigation because of the social, economic and technological changes in our world, as well as COVID-19 pandemics. One example is the availability of credit and financial innovative products (Van Aardt et al., 2009). Also, some newer endogenous factors originating from new living and working styles brought about by the recent pandemic and technological advancement should also be investigated with along with the timely impact of the COVID pandemic. Such factors and their possible relationships with financial vulnerability are reviewed and discussed below.

### Factor 1: Impact of the COVID pandemic on financial well-being

Financial well-being is “…a state of being wherein you have control over day-to-day, month-to-month finances; have the capacity to absorb financial shock; are on track to meet your financial goals; and have the financial freedom to make the choices that allow you to enjoy life” (Consumer Financial Protection Bureau, 2015, p. 5). By analysing the relevant literatures after the outbreak of COVID-19, the associated economic downturns constitute a shock leading to changes in several aspects of personal finance, including cash inflow reduction, cash outflow increase, and/or asset value and wealth reduction. Our findings are summarised below.

#### Cash inflow reduction

The causes for cash inflow reduction can be income shock (Achou et al., 2020; Hanspal et al., 2020; Jantan et al., 2020) due to unemployment, under-employment or reduction in work hours, and ineligibility/inaccessibility to the government emergency benefits (Thompson et al., 2017; Achou et al., 2020; Kempson and Evans, 2020; Roll and Despard, 2020; Round et al., 2020).

#### Cash outflow increase

Common reasons for cash outflow increase are additional expenditures such as unusual cash spendings due to pandemics, health-related expenditure, new care-related responsibilities, and/or other life altering events (Round et al., 2020). Income shock might force people to enter into debt or to increase borrowing to make ends meet, including bills and rents, or, worse, turn manageable debts into unmanageable obligations (Round et al., 2020).

#### Wealth reduction

Similarly, people might suffer from wealth shock when being forced to run down their savings as a result of adverse economic and stock market conditions, resulting in a drop-in investment value (Hanspal et al., 2020).

People who have been struggling financially before COVID-19 were more likely to experience further financial difficulties as a result of pandemic-related economic downturn (Roll et al., 2020; Round, 2020). Past literatures usually attempt to look for demographic factors and personal characteristics of the vulnerable groups, such as gender, age, family types, employment types, work sectors, education level, housing tenure, work status, marital status, etc. (Schicks, 2012; Disney and Bridges, 2016; Achou et al., 2020; Kempson and Evans, 2020; Roll and Despard, 2020; Round et al., 2020). Those research intended to identify which specific groups of people with specific features the past economic shocks caused them to be more vulnerable to financial difficulties. However, according to the more current literatures, the impacts of COVID-19 can be found more widely spread across a country or a city (e.g., Kempson and Evans, 2020). It deserves to explore what the possible new factors are, which may affect a bigger segment of people. The following are the findings from our literature survey efforts, by synthesising the new social-economic phenomenon under COVID-19 and the relevant past literatures.

### Factor 2: Digitisation of the economy since the outbreak of COVID-19

Due to technological advancement, many economic activities, such as shopping, entertainment, and payment platforms, have been digitised in recent years. COVID-19 has forced social contact to the minimal level, hence further accelerating social-economic digitisation to other aspects of daily life and financial management activities, such as working, meeting, learning, food and grocery consumption, banking, and investment (Idris, 2019; Pinochet et al., 2019; Shang et al., 2021).

Due to fierce competition amongst in marketing and social media space, digital services providers mostly focus their marketing messages to be more visible in mass media including social media and make their services more easily accessible to consumers (Idris, 2019; Moenjak et al., 2020). The new working and learning style at home not only make digital services more accessible to people, but also the related marketing messages about consumption, entertainment and credits. The triggering of purchasing or consumption can result in over-consumption and over-indebtedness (Schicks, 2013). The attitudes towards using debt-financing to support consumption of non-durable goods were found to be significantly affected by the time spent on social media (Henrik and Anton, 2020). The adverse sequence of negative consequences leading to financial problems are not sufficiently investigated (Carlsson et al., 2017; Idris, 2019).

Similarly, fintech makes people view credit as money and not as debts (Ash et al., 2018). Fueled by social influence such as influencers, celebrities and push marketing efforts of financial services companies, people with specific characteristics, especially those with higher degree of personal innovation (e.g., youths and the younger population), have higher propensity to consume credit services, resulting in problematic indebtedness (Carlsson et al., 2017). Some individuals using digital credit were found to be more likely in financial troubles as demonstrated with this population segment experiencing a higher number of loans and a higher number of incidents of selling household assets to repay loans (Wamalwa et al., 2019).

Existing literatures acknowledge this research gap regarding the understanding of how personal consumption, financial and credit behaviour may change in relation to the development of digital technology and digital society (Carlsson et al., 2017; Idris, 2019). Specifically, the vulnerability of adolescents as early adopters of digital services needs further attention (Carlsson et al., 2017; Tung, 2018). Scholars have recommended that more studies should account for digital development and investigate the new skills required for the digital consumers in credit society to further understand the conditions of personal financial behaviour as well as financial literacy education and counselling in digital society (Carlsson et al., 2017).

Pandemic-driven lockdowns have forced people to adopt more types of digital services and spend more time on social media. COVID-19 has further accelerating social-economic digitisation to other aspects of daily life because it forced social contact to the minimal level. We believe additional research should be done by broadening coverage in digital services with potentially relevant financial vulnerability, such as e-payment platforms, banking, entertainment, gambling, and investment (e.g., crypto-currencies investment). Also, with the increase in the awareness and the exposure to the marketing information and promotion message of such services, how an individual may respond to these influence and consumers’ adoption of such services bring about behavioural change in consumption, spending, use of debt and savings/investment should also be investigated in future research.

### Factor 3: Internal factors affecting financial management ability

As discussed above, digitisation of the economy and the increasingly easy availability of credit together with the flourishing innovative financial products can be taken as new external factors to fit into Van Aardt et al.’s financial vulnerability cause-and-effect chain. Nevertheless, these impacts still depend on other internal factors originating from an individual level that make a difference in terms of individual’s ability to resist temptation in a digitised society and skills in financial management. This will be discussed in the following.

#### Financial literacy

Financial literacy is a combination of awareness, knowledge, skill, attitude and behaviour necessary to make sound financial decisions and to achieve financial wellbeing (OECD/INFE, 2011; Tung, 2018). Empirical evidence has showed that this is a key factor explaining over-indebtedness (e.g., Lusardi and Tufano, 2009) and that a high level of financial literacy has a positive impact on management of personal finances and financial behaviour (Kotlikoff and Bernheim, 2001; Hilgert et al., 2003; Courchane and Zorn, 2005; Lusardi and Tufano, 2009; Capuano and Ramsay, 2011; Navickas et al., 2014). More importantly, financial literacy may improve one’s ability and capability to deal with macroeconomic shocks (Klapper et al., 2013; Paiella, 2016; Tung, 2018). In reverse, people with limited financial literacy may not make informed decisions when consuming digital credits (Wamalwa et al., 2019).

Although empirical results showed that financial literacy reduces utilisation of digital credit, better financial knowledge may not always result in behaviour change because of other constraints or interfering factors (Seiling and Shockey, 2006). Individuals can experience cognitive functions deterioration (Agarwal et al., 2009; Ansong and Gyensare, 2012). For example, people are easily influenced by the regular and repeating marketing promotions that stimulate their financial behaviour in consumption and credit card usage, offsetting the effects of financial literacy (Tung, 2019). Facing the challenges of a digitised society and more social media marketing and promotion brought about by the pandemic, the financial behaviour of people after the outbreask of COVID-19 deserves a deeper investigation by considering their financial literacy and knowledge.

In addition, there are more current literatures concerning other factors interrelating to or interfering with the effect of financial literacy and the ability on financial management within certain segment(s) of the population. Low level of financial literacy coupled with self-control problems, leading to over-indebtedness and many other financial problems such as income shocks as well as unforeseen expenses on durables (Gathergood, 2011). This is important to understand how some non-cognitive factors influencing financial behaviours (Alsemgeest, 2015). The following specifically focus on a new phenomenon after the outbreak of COVID-19.

#### Addiction to digital technology

Pandemic lockdown has created an opportunity for people to spend more time on social media and digital services such as shopping, entertainment and money management in the digital economy. Since most digital services usually involve marketing and promotion to stimulate more utilisation, impulse spending, and borrowing, individuals and their digital service usage frequency may bring about behavioural change in consumption, spending, use of debt, and savings/investment. At the individual level, the inability to resist temptation, overspending, relaxed attitudes to borrowing, together with poor money management skills are common contributing factors to problem debt (Round et al., 2020). This is especially true for heavy digital service users.

Addiction is defined as impulse dependent on a habit of a specific activity or substance use that has destructive effects on social, emotional and even financial situations (Idris, 2019). The frequency of online shopping or the time spent on social media are the risk factors and can increase the likelihood of problematic spending (Lam and Lam, 2017; Henrik and Anton, 2020).

Existing studies are very limited in investigating the effects of digital technology addiction towards over-indebtedness (Idris, 2019). Available studies have only investigated the association of problematic online shopping and financial literacy (Lam and Lam, 2017). In response to scholars’ recommendation of studying the effect of digital technology addictions towards individual financial well-being (Idris, 2019), future research can extend the scope of previous research to a broader coverage of more types of digital services and investigate how addiction to digital technology can impact financial vulnerability. Examples are digitised financial service and online entertainment such as online stock investment services, gambling platform and video-on-demand services.

### Factor 4: Changes in financial behaviour

According to Van Aardt et al.’s consumer financial vulnerability model (Van Aardt et al. 2009), the most immediate factors affecting financial vulnerability are individuals’ income fragility and expenditure, where income fragility can be further decomposed into savings related and investment related conditions. Meanwhile expenditure can be further decomposed into spending-related and debt-related conditions. All these conditions inevitably are affected by the changes in related financial behaviour as responses to either internal personal and/or external environmental situations. By systematically and separately investigate what has happened to vulnerable groups in the following four area of behavioural change, we can obtain a clear picture of factors leading to their financial difficulties, especially in the recent years with the COVID-19 pandemic (Kempson et al., 2017; Thompson et al., 2017; Achou et al., 2020; Moenjak, 2020; Roll et al., 2020; Round et al., 2020).

1. Income/saving related: Better saving habits and plan for the future can improve financial condition. Under COVID-19, what action(s) people could take to increase income or saving, or what affect their ability to increase income or saving are not well documented in existing research and therefore more investigation is required.
2. Spending related: Spend more, for example on non-essentials, and live beyond one’s means will worsen financial condition. Under COVID-19, are people forced to spend more on hygienic products or entertainment? How these affect their financial condition. More research is required.
3. Investment related: Investment is likely to increase one’s wealth. Whilst the opposite is also true. An individual who is willing to take risks tends to exhibit high risk-taking behaviour such as investing in new products and borrowing to invest (Kannadhasan, 2015; Hanspal et al., 2020). During COVID-19, possible changes in investment behaviour can be the higher frequency because of fintech, invest in new investment products due to exposing to more investment news. The understanding is insufficient from existing research.
4. Debt related: Risky borrowing, use of credit for impulsive consumption, missing or defer loans payment will worsen financial condition. In addition, some may even borrow money when experiencing financial difficulties in order to pay off other credits or other commitments (Kempson, 2002). How COVID-19 leads to changes in debt related behaviour deserves more closer study. For example, is it common for people to borrow more because of job losses or sickness? More empirical studies is necessary.

## Conclusion and future research suggestions

Although Van Aardt et al. (2009) had developed a heuristic financial vulnerability model which draws conclusions in key factors leading to financial vulnerability, with the social, economic and technological changes in recent years with the coronavirus outbreak in 2019, there are new challenges for decomposing the root causes of financial vulnerability because new factors are coming into play.

By referring to the recent literatures, we identified several factors that can be mapped into the cause-effect chain of financial vulnerability of Van Aardt et al. (2009), either as new external factors (i.e., Pandemic), internal factors (i.e., financial literacy and addiction to digital technology), or the “new version” of existing variables. These are new factors related to the current pandemic and developing technological advancement (i.e., digitisation of the economy), which in turn are bringing about new living and working styles as well as financial behavioural change (i.e., changes in financial behaviour). The emerging changes and factors were not sufficiently and holistically investigated in the existing literatures. These factors are summarised below.

Pandemic (an external factor) – COVID-19 and the associated economic downturn(s) constitute shocks leading to cash inflow reduction, cash outflow increase and/or asset value and wealth reduction. Cash inflow reduction can be the results of a combination of passive reasons, such as employers’ decision, eligibility and accessibility to the government emergency benefits, and/or family/personal reasons, such as home caring responsibilities with children staying at home as overseas domestic helpers are unable to report duty due to lockdown or flight postponement. Cash outflow increases can be caused by additional credit or debt utilisation to support extra pandemic-driven expenditure, such as facilities and equipment to support work-from-home or study-from-home, additional consumption of hygienic and protective products. The question of whether people suffer from wealth shock because of the unmanageable debts, economic downturn and/or the more volatile investment environment as topics and factors are not fully examined. In addition, given the impacts of COVID-19 was found more widely spread across a country or a city, a deeper understanding can be achieved by knowing how asset value cash inflows, and outflows changes before and after the Covid-19 so as to identify which issues that Covid-19 impact the most.

Digitisation of the economy (a “new version” of credit availability and financial product innovation) – Technological advancement coincides with the COVID-19 pandemic, which forced social contact to minimal level, to accelerate social-economic digitisation in many aspects of daily life with impacts in economic and financial management activities ranging from shopping, personal consumption, banking, payment, and credit-related application. In addition, big data is enabling digital services providers to perform targeted advertising and push marketing more aggressively to trigger more consumptions and transactions. With a high smartphone penetration rate and abundant availability of different digitised services. Peoples’ usage frequency of digitised services and awareness and exposure to related push marketing activities may increase because they have been spending more time at home to work-from-home, study-from-home arrangement, endure under-employment or even unemployment. We intent to investigate how the vulnerable groups of our population are affected by digitisation of the economy. It is important to explore how financial difficulties are caused by digitisation to potentially alter the pattern of personal consumption, financial transactions and credit behaviour due to the outbreak of COVID-19.

Financial literacy (an internal factor) – Financial literacy is a combination of awareness, knowledge, skill, attitude and behaviour necessary to make sound financial decisions and to achieve financial wellbeing. Financial literacy may improve one’s ability and capability to deal with macroeconomic shocks and reduces utilisation of digital credit. Facing the challenges of digitised society brought about by the COVID-19 pandemic, individuals’ financial competence to deal with the easier availability of digitised financial services deserves deeper investigation.

Addiction to digital technology (an internal factor) – Other factors may interfere with the effect of financial literacy and the ability to manage personal finance. Specifically, the addiction to digital technology is a phenomenon deserved attention. This is driven by the digitisation of the economy. Addiction is defined as an impulse dependent on a habit of a specific activity or substance use that can lead to destructive effects on social, emotional, and even financial conditions. The COVID-19 pandemic has created an opportunity for people to spend more time on social media and digital services to shop, to be entertained, and to manage their financial affairs. Since most digital services usually involve marketing and promotion activities to stimulate a higher levels of consumer utilisation, people may become more susceptible to impulse spending and borrowing. Essentially these impulse-driven usages may bring about behavioural change in consumption, spending, use of debt and savings/investment.

Inability to resist temptation, overspending, relaxed attitudes to borrowing, combined with poor money management skills are common contributing factors to individual debt problem. Existing studies are very limited in investigating the relational effects on digital technology addiction and people suffering from over-indebtedness. We propose to extend the scope of previous research, cover a broader range of digital services, and investigate how addiction to digital technology may impact financial vulnerability.

These two internal factors (financial literacy and addiction to digital technology) may intertwine to impact individual’s ability to resist temptation in a digitised society and skill in personal financial management.

Changes in financial behaviour – According to Van Aardt et al.’s consumer financial vulnerability model, the most immediate factors affecting financial vulnerability are individuals’ income fragility and expenditure. Income fragility can be further decomposed into savings-related and investment-related conditions. Meanwhile, expenditure can be further decomposed into spending-related and debt-related conditions. By systematically and separately investigating what behavioural changes might have happened to vulnerable groups in these four factors, we can obtain a clear picture about the causes leading to their financial difficulties in recent years, especially after the outbreak of the COVID-19 pandemic.

To conclude, the purpose of this paper is to conduct literature survey to review and consolidate the recent scattered literatures to identify some possible factors to be considered in the research related to financial vulnerability. Based on our literature survey above, four relevant issues have been identified for future research: (i) pandemic, (ii) digitisation of the economy, (iii) intertwining effect of financial literacy and addiction to digital technology, and (iv) four area of financial behavioural changes. Future research covering the above elements could deepen our understanding of how personal and environmental factors, especially the COVID-19 pandemic outbreak and socio-economic changes, interrelate to affect peoples’ financial vulnerability. One possible research plan is to focus on a particular segment of a country or city, such as people who need to seek for debt counselling services because they are in financial trouble after the outbreak of COVID-19, and study their characteristics of the above proposed issues, by questionnaire survey.

## Data availability statement

The original contributions presented in the study are included in the article/supplementary material, further inquiries can be directed to the corresponding author.

## Author contributions

All authors listed have made a substantial, direct, and intellectual contribution to the work and approved it for publication.

## Funding

This paper is produced as part of the Research Project “The characteristics of the Vulnerable Groups with Personal Financial Difficulties: The impact of COVID-19 and the implications to financial counselling needs” which has been funded by the Investor and Financial Education Council (www.ifec.org.hk). This research study was supported by Hainan Provincial Natural Science Foundation of China. The name of the project is “Research on Statistical Monitoring and Accounting of Digital Economy in Hainan Free Trade Port.” The project number is 722RC724. This paper was also supported by the Hainan College of Economics and Business (Project reference number: hnjmk2021301).

## Conflict of interest

The authors declare that the research was conducted in the absence of any commercial or financial relationships that could be construed as a potential conflict of interest.

## Publisher’s note

All claims expressed in this article are solely those of the authors and do not necessarily represent those of their affiliated organizations, or those of the publisher, the editors and the reviewers. Any product that may be evaluated in this article, or claim that may be made by its manufacturer, is not guaranteed or endorsed by the publisher.
